# Shah's Indian penile prosthesis placement after phallic reconstruction with radial forearm flap

**DOI:** 10.4103/0970-1591.38613

**Published:** 2008

**Authors:** Sujata K. Patwardhan, Rupin Shah, Vijay Kulkarni, Radheshyam R. Varma

**Affiliations:** Department of Urology, LTMMC and LTMGH, Sion, Mumbai, India; 1 Lilavati Hospital and Research Centre, Bandra, Mumbai, India

**Keywords:** Neo-phallus, radial forearm flap, rigid penile prosthesis

## Abstract

We report a successful implantation of Indian penile prosthesis after total phallic reconstruction. The differential stiffness of the Shah penile prosthesis is felt to have less potential for erosion, the most common complication of rigid prosthetic stiffening devices when used in a neo-phallus. This prosthesis is an alternative to the inflatable prosthesis in patients who choose a rigid prosthesis due to economical constraints.

## INTRODUCTION

Penile amputation is an uncommon injury resulting from self-mutilation, accidental trauma or felonious assault.[1] Phallic replacement is the treatment of choice, when the amputated segment is lost or nonviable. Microsurgical free radial forearm flap is the current mainstay of penile reconstruction therapy.[2]

We report a successful case report of placement of Shah's Indian penile prosthesis in a reconstructed penis using radial forearm flap.

## CASE REPORT

A 21-year-old, unmarried, computer operator, had self-amputated his penis by using surgical blade after self-local anesthesia with Injection lignocaine in March 2005. As per history, patient held the penis responsible for weakness and his poor physique, hence decided to self-amputate the penis. The patient discarded the amputated segment. After six months of psychiatric therapy the patient opted for phallic reconstruction and underwent a radial forearm flap neo-phallus reconstruction. Patient presented to us after one year of surgery with a request for penile prosthesis implantation as he was planning to get married.

**Figure 1:**
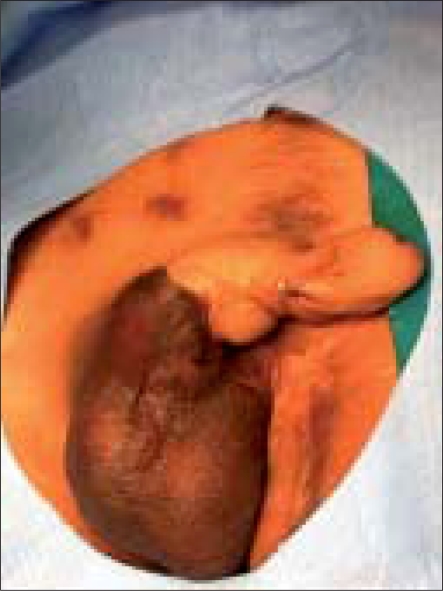
Preoperative photograph

On examination, [Figure 1] 1 inch of fibrotic corpora was palpable at the base of the penis and the rest of the palpable corpora (1 inch) was supple. He had mid-penile hypospadias, which could be calibrated up to 12 French. The neo-phallus had sensations of light touch and vibration. A Biomedical Biosthesiometer was not used to document tactile sensibility of the penis. The scrotal sac and both the testes were normal. Ultrasound of the perineum and abdomen revealed no evidence of fibrosis in the posterior crura and his post-void residual was nil. Psychiatric referral ruled out any active psychiatric illness.

**Figure 2:**
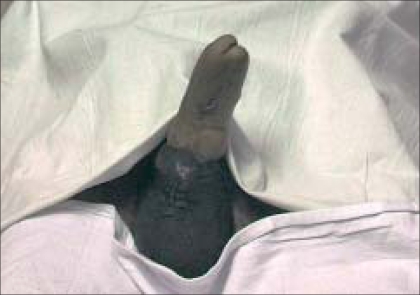
Postoperative photograph

After vertical penoscrotal skin incision, a cruciate incision was taken on the fibrotic anterior tip of the crura. The fibrosis of the anterior corpora was difficult to dilate, but was eventually possible using sharp dissection. This was then continued into the subcutaneous tissue in the forearm flap which was carefully dilated. A distal cushion of tissue measuring 1.5 cm was preserved. The posterior crura were serially dilated. WH 09 the Shah Indian penile prosthesis – (model WH09) - was selected for insertion. Length of prosthesis inserted was 17 cm with both sleeves peeled off to reduce the diameter of the implant from 13 mm to 9 mm (another unique feature of the Shah implant). Implant was not fixed to the any part of the neo-phallus or corpora. Urethral catheter was kept for seven days postoperatively. Patient was given intravenous antibiotics for seven days followed by oral cephalosporins for the next 14 days. Postoperatively, the patient had no complications [Figure 2]. On five months follow-up patient had normally functioning prosthesis with no evidence of any graft infection/erosion.

## DISCUSSION

Penile injury is a rare injury resulting from self-mutilation, accidental trauma or felonious assault. Though there are various methods of replacing nonviable penile tissue, microsurgical free radial forearm flap is the mainstay of penile replacement therapy.[2]

The goal of achieving reliable phallic rigidity has remained a challenge to surgeons till date. Bogoraz and Frumken first suggested the use of a stiffener and reported initial satisfaction with costal cartilage.[3] Goodwin and Scott used autogenous costal cartilage rods to substitute the corpora cavernosa.[3] Long-term results were not reported. Chang and Hwang incorporated a cartilage stiffener that could be removed to provide space for future prosthesis placement[2]. A successful osteocutaneous radial forearm flap using a portion of radial bone was reported with a 10-month follow-up.[3]

Autogenous materials like cartilage and bone fail to remain rigid because of resorption and do not provide a normal erectile angle.

Later, surgical innovations led to use of acrylic and silicon rods. The problems with their use were erosion, spontaneous dislodgment and poor concealibility. Jordan *et al.*, created a neotunica with Goretex graft which acts a sleeve surrounding the actual implant.[4] The inflatable cylinder is ensheathed in the Gortex Sleeve.

The main problems in a rigid penile implant relate to ischemic damage due to chronic pressure and shear forces. This in turn leads to the dreaded complication of erosion. The Indian penile prosthesis is a differential rigidity implant and is made of implantable grade silicon of varying softness. The tip of the implant is soft and made of 25 shore A silicon – this minimizes the pressure on the tip of the neo-phallus and reduces the chances of pressure necrosis and erosion. The anterior shaft of the implant is 75 shore A, ensuring adequate stiffness. The central part of the implant acts as a hinge and is made of 25 shore A silicon - this flexibility at the hinge further helps reduce the axial pressure exerted by the implant. The posterior zone of the implant is of 50 shore A silicon and is narrow in diameter, allowing it to be placed even in fibrous crura.

In patients who cannot afford an inflatable device worth Rs. 2,25 lacs the Shah Indian penile prosthesis is an economical alternative. The malleable hinge and soft tip have less potential for erosion as compared to the malleable implant.

Long-term follow-up of this patient when he becomes sexually active will further validate the use of the Shah implant in a neo-phallus.

## CONCLUSION

Implantation with Indian penile prosthesis is a safe, affordable option in the Indian scenario if the patient cannot afford imported rigid or inflatable penile prosthesis.

## References

[1] Jezior JR, Brady JD, Schlossberg SM (2001). Management of penile amputation injuries. World J Surg.

[2] Chang TS, Hwang WY (1984). Forearm flap in one-stage reconstruction of the penis. Plast Reconstr Surg.

[3] Levine LA, Zachary LS, Gottliee LJ (1993). Prosthesis placement after total phallic reconstruction. J Urol.

[4] Jordan GH, Alter GJ, Gilbert DA, Horton CE, Devine CJ (1994). Penile prosthesis implantation in total phalloplasty. J Urol.

